# Importance of correcting alar base ptosis during primary cleft lip repair

**DOI:** 10.20407/fmj.2022-014

**Published:** 2022-10-28

**Authors:** Maki Inukai, Yoshikazu Inoue, Yoshimi Sano, Satoko Onishi, Takayuki Okumoto, Ichiro Uyama

**Affiliations:** 1 Department of Plastic and Reconstructive Surgery, Fujita Health University, School of Medicine, Toyoake, Aichi, Japan; 2 Division of Pediatric Dentistry and Orthodontics, Department of Plastic Surgery, Fujita Health University, School of Medicine, Toyoake, Aichi, Japan; 3 Department of Plastic and Reconstructive Surgery, Ehime Rosai Hospital, Niihama, Ehime, Japan; 4 Department of Advanced Robotic and Endoscopic Surgery, Fujita Health University, School of Medicine, Toyoake, Aichi, Japan

**Keywords:** Cleft lip and palate, External rhinoplasty, Millard method, Cleft lip repair, Alar base ptosis

## Abstract

**Objectives:**

Until 1999 at our hospital, primary cleft lip repair was performed by the straight-line method and external rhinoplasty was performed by the inverted trapezoidal suture method with bilateral reverse-U incisions for children with cleft lip and palate. Subsequently, repeated surgical corrections of the external nasal morphology became necessary during the growth period, often with unsatisfactory results because repeated external rhinoplasty results in a stronger scar contracture. From 2000 to 2004, we performed external rhinoplasty after patients had stopped growing; however, delaying surgery created a psychological burden for patients. Therefore, since 2005, we have focused on improving alar base ptosis and forming the nostril sill during the primary surgery. This study was performed to subjectively and objectively evaluate whether the current surgical method or the earlier technique produces a better treatment outcome.

**Methods:**

We subjectively and objectively evaluated alar base asymmetry after primary cleft lip repair but before bone grafting for alveolar cleft repair. For the objective evaluation, we measured the angle of alar base ptosis in frontal view photographs taken at the age of 6 or 7 years in patients who underwent repair before 1999 (Group A) and after 2005 (Group B).

**Results:**

The median angle was 2.75° in Group A and 1.50° in Group B, demonstrating a significant difference (P=0.04).

**Conclusions:**

The current surgical method, which reflects our focus on improving alar base ptosis and forming the nostril sill, subjectively and objectively improved the external nasal morphology.

## Introduction

**Figure 1:**
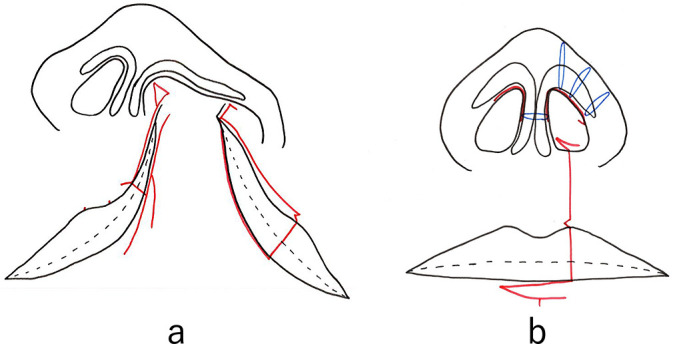
Straight-line method for repair of cleft lip and palate (a) Incision lines for the straight-line method. The schematic shows the straight-line method for cleft lip repair before surgery. This method was used at our hospital until 1999. Red line: preoperative marking. (b) Completed wound closure (red line) with additional sutures on medial crura and alar transfixion sutures (blue line) for the straight-line method. The schematic shows the straight-line method for cleft lip repair after suturing is completed. Blue line: intercartilaginous suture of the greater alar cartilage; red line: suture line.

Over the years, the approach to surgical repair of cleft lip and palate has changed with the aim to improve patients’ facial appearance. Until 1999 at our hospital, surgical repair of cleft lip and palate was performed from 2 weeks after birth by the straight-line method, and external rhinoplasty was performed by the inverted trapezoidal suture method through bilateral reverse-U incisions.^1^ To minimize postoperative scarring and achieve symmetry with the bilateral philtrum, we used the straight-line method with a very small triangular flap (Figure 1a). The advantage of this method was that it avoided the unnatural scar across the white lip formed by the Millard method.^2–4^ However, the technique focused on minimizing postoperative scarring and not on forming the nostril sill or rolling the alar base inward. In addition, external rhinoplasty was simultaneously performed by making an inverted U-shaped incision at the nostril margin on the affected side; if the external nasal deformity was severe, an incision was also made at the nostril margin on the unaffected side, and the subcutaneous skin over the bilateral greater alar cartilage and lateral nasal cartilage was dissected. Additional intercartilaginous sutures were added as needed until a satisfactory external nasal morphology was achieved (Figure 1b).

The disadvantage of the straight-line method was that it failed to create a nostril sill because of alar base ptosis, and the external rhinoplasty focused only on the nostril rim shape when looking up at the nose from below. Furthermore, the results were not satisfactory, and in many cases external rhinoplasty had to be repeatedly performed during the growth period, resulting in a high degree of scar contracture that ultimately made correction difficult. Therefore, beginning in 2000, we delayed external rhinoplasty until after patients had finished growing. The advantage of delaying the operation was that no postoperative scarring of the external nasal components occurred, the nasal cartilage retained its shape, and there was no need to consider changes due to growth. However, the preoperative external nasal deformity represented a psychological burden for adolescents. To reduce this burden, we decided to create an external nasal shape during primary cleft lip repair that would be acceptable to the patient and others.

**Figure 2:**
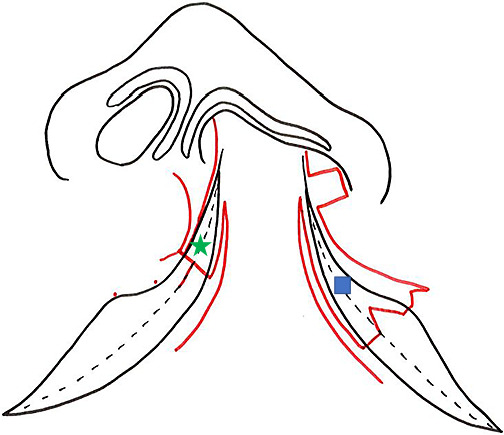
Incision lines for Millard variation plus small triangular flap method The schematic shows the Millard variation plus small triangular flap method for primary cleft lip repair. We have used this approach since 2005. Red line: preoperative marking; green star and blue square: cleft margin mucosal flap for the lining to form the nostril floor.

**Figure 3:**
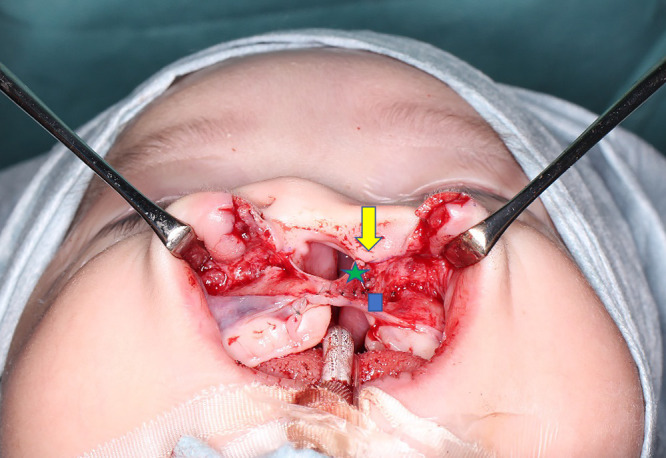
Completion of nostril floor reconstruction This photograph shows the cleft margin flap on the greater side (green star) sewn to the high point of the piriform margin on the lesser side margin (yellow arrow) in the Millard variation plus small triangular flap method. We consider it important to elevate the lateral wall of the nasal vestibule well above the piriform margin and suture the cleft margin flap on the greater side (green star) used for lining the nasal floor as high as possible above the piriform margin. The cleft margin flap on the lesser side (blue square) is sutured to the bottom of the cleft margin flap on the greater side (green star).

**Figure 4:**
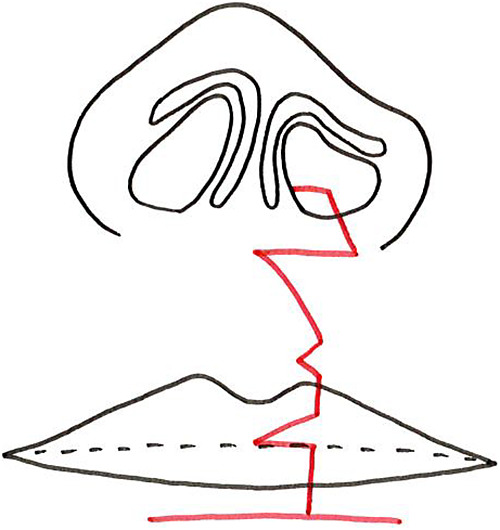
Completed wound closure (red line) for Millard variation plus small triangular flap method The schematic shows the Millard variation plus small triangular flap method after completion of suturing. Red line: suture line.

Consequently, in 2005 we began to focus on optimizing frontal view symmetry by improving alar base ptosis and forming the nostril sill during the primary repair.^5^ We started performing cleft lip repair based on the Millard variant method plus the small triangular flap technique in 2000 (Figure 2).^2–4,6–8^ We have continued to use this surgical approach since 2005, but we now elevate the lateral wall of the nasal vestibule well above the piriform margin and suture the cleft margin flap on the greater side used for lining the nasal floor as high as possible above the piriform margin (Figure 3). Next, we sufficiently separate the external nasal component and the lip component, roll the alar base of the external nasal component inward, and suture the nostril floor to ensure that the alar base and nasal floor remain elevated. In contrast to the nasal alar component, the lip component is rotated inward and downward so that the shortened white lip is lengthened without being pulled by the external nasal component. For the external nasal component, we roll the alar base and create the nostril sill without involving the nasal cartilage (Figure 4).

We currently perform primary cleft lip repair after the age of 1 month. From the time of the initial consultation to the time of surgery, we instruct parents to use tape to pull the cleft lip to the left and right and to insert a nasal retainer to correct the external nasal deformity. We also introduce a palatal plate in patients with a cleft palate.

To date, we have not analyzed the differences in outcomes between the surgical approaches to cleft lip repair and external rhinoplasty that we used until 1999 and after 2005. Therefore, in addition to subjectively comparing patients’ appearance, we performed this retrospective study to objectively compare the external nasal morphology after treatment with the two surgical approaches.

## Methods

We subjectively compared four patients treated during the years up to and including 1999, when external rhinoplasty was performed at the same time as the primary surgery, with four patients treated after 2005, when the surgical technique was improved to obtain a symmetrical external nasal form in the frontal view. In addition, we objectively compared 21 consecutive patients who underwent surgery from January 1997 to November 1998 (Group A), when the surgeon’s technique was considered the most stable, with 23 consecutive patients who underwent surgery from January 2005 to September 2008 (Group B). All patients had a unilateral complete cleft lip, alveolus, and palate or a unilateral complete cleft lip and alveolus, and all operations were performed by the same surgeon. The patients were evaluated before bone grafting for alveolar cleft repair.

**Figure 5:**
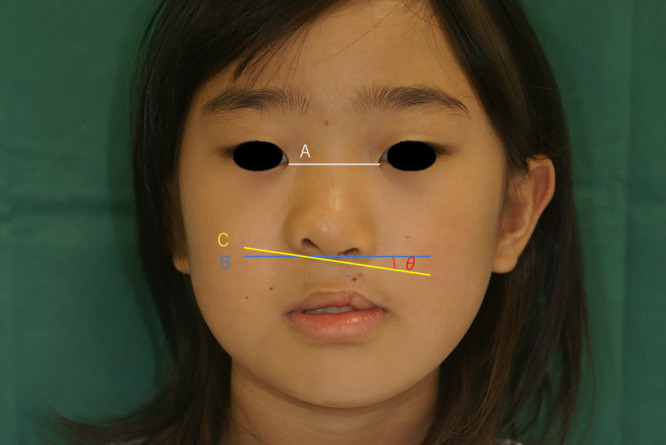
Measurement of alar ptosis Line A connects the bilateral medial canthi, line B passes through the alar base on the cleft side and is parallel to line A, and line C connects the alar bases on the non-cleft side and cleft side. Angle (θ) is the angle between lines B and C.

Alar base ptosis was assessed in frontal view photographs taken at the age of 6 or 7 years by measuring the angle (θ) between a line drawn through the alar base on the non-cleft side (line B) (which was parallel to a line connecting the bilateral medial canthi, line A) and a line drawn between the two alar bases on the non-cleft side and the cleft side (line C) (Figure 5). Patients who had undergone secondary correction of the lip or nose after the primary surgery were excluded from the study.

This retrospective study was approved by the ethics committee of Fujita Health University (No. HM21-444). Consent for participation in this study was obtained by an opt-out method.

### Statistical analysis

The statistical analysis was performed with the statistical software R (3.5.2). Means with standard deviations (SDs) and medians were calculated for each group, and the groups were compared by the Mann–Whitney test. The level of statistical significance was set at α=0.05.

## Results

**Figure 6:**
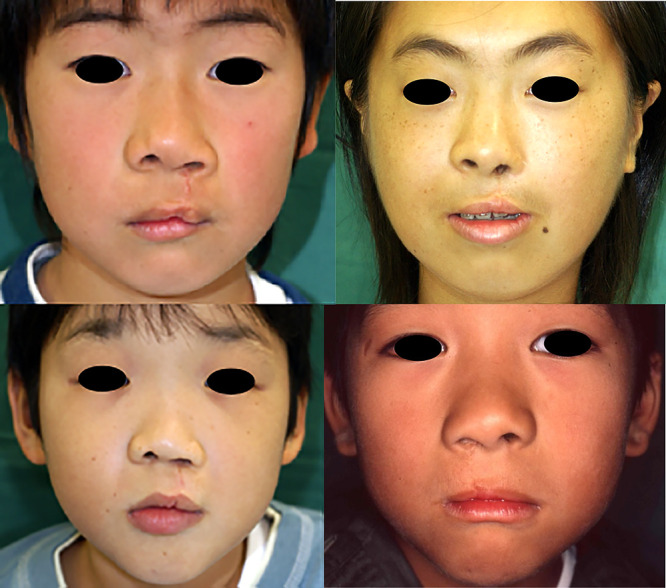
Patients who underwent surgery using straight-line method These photographs show patients aged 7 years who underwent cleft lip repair by the straight-line technique until 1999. The asymmetry of the alar base is clearly visible.

**Figure 7:**
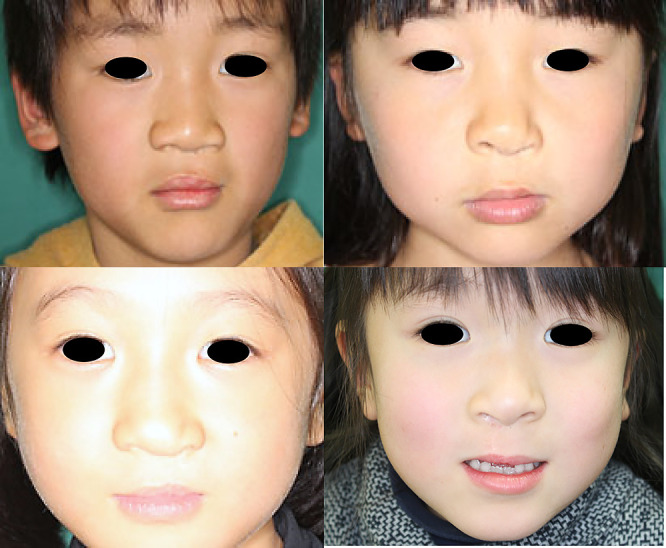
Patients who underwent surgery using Millard variation plus small triangular flap method These photographs show patients aged 7 years who underwent modified cleft lip repair from 2005 onward.

The subjective comparison of the external nasal morphology in children who underwent operations before 1999 (Figure 6) and after 2005 (Figure 7) showed a better appearance of the alar base in many patients in the latter group.

Group A comprised 21 patients (11 boys and 10 girls), 5 with cleft lip on the right side and 16 on the left side. Group B comprised 23 patients (13 boys and 10 girls), 11 with cleft lip on the right side and 12 on the left side.

**Figure 8:**
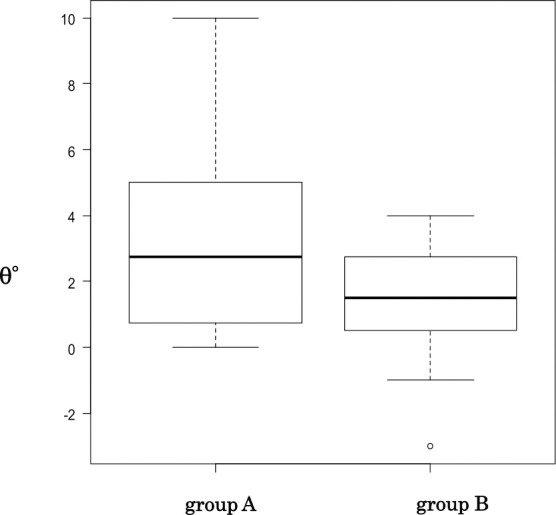
Box-and-whisker plot Box-and-whisker plot of the angle (θ) between the line that runs through the alar base on the non-cleft side (line B in Figure 5; this line is parallel to the line connecting the bilateral medial canthi; i.e., line A in Figure 5) and the line drawn between both alar bases on the non-cleft side and cleft side (line C in Figure 5). The angle was used as the degree of alar base ptosis. Group A comprised children who underwent cleft lip repair by the straight-line method. Group B comprised children who underwent cleft lip repair by the Millard variation plus small triangular flap method. The median angle was significantly greater in Group A (2.75°; range, 0°–10°) than in Group B (1.50°; range, –3° to 4°) (P=0.04).

The median angle was significantly greater in Group A (2.75°; range, 0°–10°) than in Group B (1.50°; range, –3° to 4°) (P=0.04). The data in Group A showed more variation, as evident from the larger SD and as shown by a box-and-whisker plot (Figure 8).

## Discussion

Until 1999, at the time of primary cleft lip repair surgery, we used the straight-line method and simultaneously performed the nasal cartilage suture method with reverse-U incisions on both sides of the nostril margins to repair external nasal deformities. Ideally, the primary cleft lip repair surgery alone should produce satisfactory results. However, when we evaluated the long-term postoperative outcome of this technique, we found that the nose was generally shorter, alar base ptosis was present on the affected nostril sill, and the apex of Cupid’s bow was steeper on the affected than unaffected side (Figure 6).^5,9,10^ Therefore, it is important to prevent irreparable deformities during primary cleft lip repair.^11,12^

Our results showed that the change in surgical technique improved alar base ptosis. Our results also indicated that it is difficult to achieve a stable outcome with the straight-line method as evidenced by the larger SD in Group A, which showed large variation in the degree of ptosis after simultaneous rhinoplasty and cleft lip repair.

McComb and Coghlan^13^ pointed out that in patients treated with the straight-line method, the external nose remains short in the long-term postoperative course. Among patients with complete cleft lip and palate, the nasal morphology of those treated with the straight-line method is characterized by alar base ptosis, deficiency of the nostril sill, and deviation of the base of the columella. Furthermore, the entire external nasal pyramid becomes twisted, and almost all patients require revision surgery after the primary external rhinoplasty. In addition, the nasal cartilage that is detached during the primary external rhinoplasty is indistinguishable from the scar, and the nasal cartilage itself loses its shape and does not support the nasal morphology. As the patient grows, the tendency to develop a short nose becomes stronger because of scar contracture, which is very difficult to correct. We noticed that patients who underwent primary surgery at other hospitals and were transferred to our hospital without any previous rhinoplasty had clearly visible greater alar cartilages, and we found that simple intercartilaginous sutures were sufficient to improve the external nasal morphology. Based on our long-term experience, in 2000 we stopped performing external rhinoplasty during the growth period.

In the straight-line method, the lip and nasal ala move as one mass, causing the alar base to droop on the cleft side and making it difficult to form the nostril sill. In contrast, in the method we currently use (Millard variation plus small triangular flap method), the external nasal component and lip component are separated, and the external nasal component is formed by rolling the nasal ala upward and inward to define the position of the alar base while the lip is rotated downward and inward to create sufficient white lip length. As a result, a nostril sill is also formed. McComb and Coghlan^13^ also stated that the symmetry of the position of the alar base is an important point in evaluating the appearance of the external nose. We believe that it is important to sufficiently elevate the lateral wall of the nasal vestibule above the piriform margin and to suture the cleft marginal red-lipped mucosal flap on the greater segment used for lining the nasal floor as high as possible above the piriform margin. Noordhoff et al.^11^ and Salyer^14^ also emphasized the importance of elevating the nasal outer vestibular wall and the alar base during the primary repair. In such cases, the part of the orbicularis oculi muscle that abnormally stops at the alar base on the affected side must be separated to ensure sufficient mobilization. It is also important to secure the suture fixation between the upper part of the orbicularis oculi muscle on affected side and the subcutaneous tissue of the anterior nasal spine to prevent postoperative retroversion. In this study, we subjectively and objectively compared the surgical method we have been using since 2005 with the method we used until 1999 and confirmed that the current method improves both alar base ptosis and the external nasal morphology.

In conclusion, we developed a surgical method that achieves an acceptable external nasal shape without external rhinoplasty at the time of primary cleft lip repair. There is no global standard surgical technique for cleft lip. However, many hospitals have opted for a technique based on the Millard method. Our experience with the unnatural linear scar toward the nasal cavity that occurs after use of the straight-line method led us to consider the importance of also creating a nostril sill. Therefore, we adopted the Millard method, which allows for formation of the nostril sill. Moreover, we developed a further alteration of the Millard method: the Millard variation plus small triangular flap method. In this study, we examined the postoperative external nasal morphology and found that our surgical method subjectively and objectively improves the alar base ptosis on the affected side, indicating that the change in surgical technique was effective in improving the external nasal morphology. We believe that our current method allows us to improve the external nasal morphology with minimal surgery, without scarring of the nasal cartilage, and without the psychological burden of delaying external rhinoplasty until after patients have finished growing. In fact, we feel that major modifications were largely no longer necessary by the end of the growth period because few growth-related changes occur in the external nasal morphology.
